# Development of a novel risk model to predict CRPC progression following IMRT: Implications for tailoring treatment intensity

**DOI:** 10.1002/bco2.70074

**Published:** 2025-09-07

**Authors:** Takashi Ogata, Rihito Aizawa, Hiroyasu Abe, Takayuki Goto, Kiyonao Nakamura, Yuki Kita, Takayuki Sumiyoshi, Kaoru Murakami, Kei Mizuno, Satoshi Morita, Takashi Kobayashi, Takashi Mizowaki

**Affiliations:** ^1^ Department of Radiation Oncology and Image‐Applied Therapy, Graduate School of Medicine Kyoto University Kyoto Kyoto Japan; ^2^ Department of Biomedical Statistics and Bioinformatics, Graduate School of Medicine Kyoto University Kyoto Kyoto Japan; ^3^ Department of Urology, Graduate School of Medicine Kyoto University Kyoto Kyoto Japan; ^4^ Department of Radiation Oncology Kyoto City Hospital Kyoto Kyoto Japan

**Keywords:** castration‐resistant, intensity‐modulated radiation therapy, prostate cancer, risk model, young age

## Abstract

**Objectives:**

To develop a novel risk score (RS) model to predict the probability of progression to castration‐resistant prostate cancer (PCa) (CRPC) after intensity‐modulated radiation therapy (IMRT) for patients with high‐ and very high‐risk PCa according to the National Comprehensive Cancer Network (NCCN) risk classification, since accurate prediction of the clinical outcome of definitive radiation therapy for patients with high‐ and very high‐risk PCa remains challenging due to its heterogeneity.

**Materials and Methods:**

We conducted a retrospective review of 600 patients with high‐ and very high‐risk PCa treated with IMRT at our institution. They were randomly divided into discovery (n = 300) and validation (n = 300) cohorts. A predictive RS model was created using a dataset from the discovery cohort based on the following parameters: T‐stage, Gleason score, prostate‐specific antigen and age at initiation of IMRT. The model was internally validated using a dataset from the validation cohort. RS was calculated using multivariable Cox regression analysis, and patients were categorized into low‐risk, intermediate‐risk or high‐risk based on the value.

**Results:**

The median follow‐up period of the 600 patients was 9.1 (IQR: 6.1–11.6) years. The 10‐year CRPC‐free rates for low‐, intermediate‐ and high‐risk categories were 100.0, 90.4 and 61.4% in the discovery cohort, respectively (p < 0.001). Such differences were reproduced in the validation cohort. Specifically, those rates for low‐, intermediate‐ and high‐risk categories were 96.4, 90.7 and 74.8% in the validation cohort, respectively (p < 0.001). Harrell's C‐index for this model was 0.692, being higher than that of the NCCN risk classification (0.617).

**Conclusion:**

This RS model provided useful information to enable tailoring of the treatment intensity for this heterogeneous population.

**Abbreviations**
A‐HT: Adjuvant‐hormonal therapy
ARSIs: Androgen receptor signalling inhibitors
BF: Biochemical failure
CI: Confidence interval
CRPC: Castration‐resistant prostate cancer
CT: computed tomography
GS: Gleason score
EBRT: External‐beam radiation therapy
HR: Hazard ratio
HT: Hormonal therapy
IGRT: Image‐guided radiation therapy
IMRT: Intensity‐modulated radiation therapy
IQR: Interquartile range
LH‐RH: Luteinizing hormone‐releasing hormone
NA‐HT: Neoadjuvant‐hormonal therapy
NCCN: National Comprehensive Cancer Network:
OS: Overall survival
PCa: Prostate cancer
PSA: Prostate‐specific antigen
PSMA‐PET/CT: prostate specific membrane antigen targeted positron emission tomography/computed tomography
RS: Risk score
TR: Testosterone recovery

## 1 INTRODUCTION

High‐dose intensity‐modulated radiation therapy (IMRT) in combination with hormonal therapy (HT) is one of the standard treatments for patients with localized and locally advanced prostate cancer (PCa).^1^, ^2^, ^3^, ^4^ Technological advances, such as image‐guided radiation therapy (IGRT), have helped realize excellent disease control and safety.^1^, ^5^

However, patients with high‐ and very high‐risk PCa according to National Comprehensive Cancer Network (NCCN) risk classification comprise a heterogenous population that exhibits single to multiple risk factors, and a subsequent wide range of clinical behaviour is typically observed even within the given definitions.^6^ Although numerous risk classifications have been developed to help predict a prognosis,^2^, ^7^, ^8^ the accuracy of categorizations is still unsatisfactory, as most previous investigations included risk classifications or nomograms mainly designed based on a combination of the only three established risk factors: clinical T‐stage, Gleason score (GS) and prostate‐specific antigen (PSA).^7^, ^8^ In addition, most endpoints of those risk models were basically set as biochemical failure (BF), which is currently considered unsuitable for use as an early endpoint to substitute for overall survival (OS).^9^ Although novel risk models based on molecular biomarkers or genes, such as the Decipher genomic classification tool,^10^ have been developed to identify the aggressiveness of tumours, those models are considered difficult to routinely apply in daily clinical practice due to the need for additional genomic tests.

We previously investigated factors that could be used to predict castration‐resistant PCa (CRPC) progression following high‐dose IMRT for nonmetastatic PCa.^11^ As a result, younger age was identified as an independent predictive factor for CRPC progression. Therefore, in the current study, we aimed to develop a novel risk score (RS) model by combining this promising factor with established risk factors, as well as setting the endpoint as CRPC progression, which is considered a reasonable surrogate for survival outcomes.^12^

## 2 MATERIALS AND METHODS

In accordance with the tenets of the Helsinki Declaration, this study was approved by the institutional ethical review board (approval number: R1048–2, approval date: October 30, 2023). Since the study was performed retrospectively, written informed consent was not obtained. Instead, an opt‐out form was made available on the website of our institution, and those who refused were excluded.

### 2.1 Data Collection and Study Cohorts

We reviewed our institutional database of PCa cases and searched for patients meeting the following criteria: *(1)* histologically confirmed prostate adenocarcinoma categorized as high‐ or very high‐risk based on NCCN risk classification version 2024.4^2^; *(2)* receiving definitive IMRT with a dose of ≥ 70 Gy in conventional fraction and short‐term neoadjuvant‐HT (NA‐HT) between September 2000 and July 2017 at our institution. Patients who irregularly received long‐term NA‐HT (cut‐off: 18 months) or adjuvant HT (A‐HT), or who developed CRPC during NA‐HT prior to IMRT, were excluded. As a result, a total of 608 patients met the inclusion criteria. Among them, eight patients were excluded for the following reasons: previous definitive treatment for PCa in six and lost to follow‐up just after the completion of IMRT in two. Therefore, a total of 600 patients were included in the analysis. The details of our treatment protocol regarding IMRT, IGRT and HT (including salvage treatment) were reported previously.^13^, ^14^, ^15^, ^16^ Briefly, short‐term NA‐HT (median: 7.3 months) was applied to all patients, and A‐HT was not applied. Instead, salvage HT was initiated in the early phase after BF. Specifically, salvage HT was initiated when PSA levels exceeded 4.0 ng/ml in a monotonically increasing manner to eliminate false failure cases (BF without continuous PSA elevation). As NA‐HT, combined androgen blockade with a luteinizing hormone‐releasing hormone (LH‐RH) analogue and antiandrogenic agent was performed. However, there were variations in the treatments because some patients received NA‐HT at other institutions and others with liver dysfunction or special requests received the LH‐RH analogue only. In addition, from June 2011, we selectively added 2‐year A‐HT to short‐term NA‐HT and IMRT for LAPCa patients with all of the following unfavourable risks: clinical T3‐4N0M0, GS sum ≥8 and PSA ≥ 30 ng/ml, based on our previous interim analysis of outcomes. Patients who received long‐term A‐HT under this protocol (n = 44) were also excluded,^17^ as described in the inclusion criteria, because the ADT method was markedly different. For the same reason, 10 patients who had received adjuvant ADT at other institutions were also excluded. Initial evaluations included needle biopsies (usually ≥ 8 cores), digital rectal examinations, transrectal ultrasonography, computed tomography (CT), magnetic‐resonance imaging and bone scintigraphy. Prostate‐specific membrane antigen‐targeted positron emission tomography/computed tomography (PSMA‐PET/CT) was not used for the initial diagnosis in any case. Cores from prostate needle biopsy and radiographical imaging examinations that had been performed at other institutions were re‐reviewed at our institution.

### 2.2 Statistical Analysis: Endpoint

The endpoint of this RS model was the CRPC progression‐free rate. Time zero was set at the initiation of IMRT. The CRPC progression‐free rate was calculated using the Kaplan–Meier method, in which other causes of death in the absence of CRPC progression were censored at the time of the last visit or when clinical data were available. Progression to CRPC was defined as the earliest timing of the following: *(1)* increase of PSA levels above 1.0 ng/ml from the nadir under castration levels of testosterone (<50 ng/dl) (definition of Prostate Cancer Working Group 3^18^) or during HT (if testosterone was not evaluated at appropriate timing); *(2)* change in HT by physician's judgement due to disease progression, even though the increase of PSA levels was under 1.0 ng/ml from the nadir; or *(3)* clinical failure during HT detected on radiographical imaging examinations such as CT scan, magnetic resonance imaging and bone scintigraphy. PSA elevation during the off‐period of intermittent HT was not regarded as an event of CRPC progression. To establish this novel RS model, the following four reported risk factors were selected: age at initiation of external‐beam radiation therapy (EBRT),^11^, ^19^, ^20^ and the three established risk factors of T‐stage, GS and pretreatment PSA.

### 2.3 Statistical Analysis: Model Development

As the first process, cut‐off values of the four reported risk factors: T‐stage, GS, PSA and age at IMRT initiation were determined. The cut‐off values of T‐stage, GS and PSA were selected with reference to those used in NCCN risk classifications.^2^ The cut‐off values of the age at IMRT initiation were determined based on the results of univariate and multivariable Cox proportional regression analysis for all cases and the distribution of patients for each age. The validity of the determined cut‐off values was evaluated using univariate and multivariable Cox proportional hazards models.

As the second process, all patients (n = 600) were randomly divided into discovery (n = 300) and validation (n = 300) cohorts at a one‐to‐one ratio. Random selection was executed by the SURVEYSELECT procedure in SAS 9.4.

As the third process, using data from the discovery cohort (n = 300), coefficients of each risk category created in the first process were calculated using multivariable Cox regression analysis, and then 1 score each was assigned to those risk categories with every 0.25 increase in the coefficient (rounding off to the nearest integral number). For example, if the coefficient was 0.79, the assigned score of the category was 3 because 1 score each was assigned for every increment of 0.25 in the coefficient. The sum of the scores from the four risk factors was defined as “RS” for CRPC progression of the current risk model. RS of each patient in the discovery cohort was calculated in order to divide them into the following three risk categories: low‐, intermediate‐ and high‐risk, in which the cut‐off of RS for the three risk categories was defined based on the inflexion point of the hazard ratio (HR) of CRPC progression of patients assigned to each score group.

As the fourth process, patients in the validation cohort (n = 300) were divided into low‐, intermediate‐ and high‐risk groups. CRPC‐free rates of each risk group were calculated using the Kaplan–Meier method, and differences were compared using the log‐rank test. Harrell's C‐index was employed to evaluate the accuracy of the RS model. As an additional investigation, to assess the accuracy of our RS model, Harrell's C‐index from our RS model was compared with Harrell's C‐index calculated based on NCCN risk classification obtained for the validation cohort.

Statistical analyses were performed using SAS 9.4 (SAS Institute, Inc., Cary, NC, USA) and R version 4.1.3 (The R Foundation for Statistical Computing, Vienna, Austria).

## 3 RESULTS

### 3.1 Patient and Treatment Characteristics

Characteristics of the 600 patients (discovery and validation cohorts) are shown in Table 1. The median age of patients was 71 years old (interquartile range [IQR]: 66–75) at IMRT initiation. Approximately two‐thirds of the patients had ≥T3a disease, a quarter of the patients showed GS of ≥4 + 5 and the median initial PSA level was 20.1 (IQR: 10.9–35.8) ng/mL, respectively. The median prescribed dose to the prostate was 78 (IQR: 74–78) Gy with 2 Gy per fraction, and prophylactic pelvic nodal irradiation was performed in 7.8%. The median duration of NA‐HT was 7.3 (IQR: 6.1–8.8) months. No patients received A‐HT or androgen receptor signalling inhibitors (ARSIs) in the setting of castration‐sensitive PCa. The median follow‐up period was 9.1 (IQR: 6.1–11.6) years.

**TABLE 1:** Patient and treatment characteristics.

	discovery cohort (*n* = 300)	validation cohort (*n* = 300)	Total (*n* = 600)
Clinical characteristic			
Age (year‐old), *n* (%)			
≤ 65	75 (25.0)	65 (21.7)	140 (23.3)
> 65 and ≤ 75	148 (49.3)	166 (55.3)	314 (52.3)
> 75	77 (25.7)	69 (23.0)	146 (24.3)
Median (IQR) (year‐old)	71 (66–76)	71 (66–75)	71 (66–75)
T stage, *n* (%)			
≤ T2c	116 (38.7)	106 (35.3)	222 (37.0)
T3a	137 (45.7)	142 (47.3)	279 (46.5)
≥ T3b	47 (15.7)	52 (17.3)	99 (16.5)
GS, *n* (%)			
≤ 7 (3 + 3, 3 + 4, and 4 + 3)	107 (35.7)	115 (38.3)	222 (37.0)
4 + 4 and 3 + 5	122 (40.7)	106 (35.3)	228 (38.0)
4 + 5	50 (16.7)	62 (20.7)	112 (18.7)
primary 5 (5 + 4 and 5 + 5)	21 (7.0)	17 (5.7)	38 (6.3)
PSA (ng/mL), *n* (%)			
≤ 20	149 (49.7)	150 (50.0)	299 (49.8)
> 20	151 (50.3)	150 (50.0)	301 (50.2)
Median (IQR)	20.1 (11.1–35.3)	20.1 (10.7–37.3)	20.1 (10.9–35.8)
NCCN risk classification, *n* (%)			
High‐risk	114 (38.0)	120 (40.0)	234 (39.0)
Very high‐risk	186 (62.0)	180 (60.0)	366 (61.0)
Duration of NA‐HT (month)			
Median (IQR)	7.3 (6.2–8.8)	7.3 (6.0–8.7)	7.3 (6.1–8.8)
IMRT dose, *n* (%)			
78 Gy	204 (68.0)	207 (69.0)	411 (68.5)
74 Gy	74 (24.7)	68 (22.7)	142 (23.7)
70 Gy	22 (7.3)	25 (8.3)	47 (7.8)
Radiation field, *n* (%)			
PORT	276 (92.0)	277 (92.3)	553 (92.2)
WPRT	24 (8.0)	23 (7.7)	47 (7.8)
Follow‐up periods (year)			
Median (IQR)	8.5 (6.2–11.4)	9.4 (6.1–11.7)	9.1 (6.1–11.6)

Abbreviations: IQR, interquartile range; GS, Gleason score; PSA, prostate‐specific antigen; NCCN, the National Comprehensive Cancer Network; NA‐HT, neoadjuvant hormonal therapy; IMRT, intensity‐modulated radiation therapy; PORT, prostate‐only radiation therapy; WPRT, whole‐pelvic radiation therapy.

### 3.2 Model Development

As the first process, T‐stage, GS and PSA were categorized based on the cut‐off of the existing risk classifications, as follows: T‐stage (≤ T2c vs. T3a vs. ≥ T3b), GS (≤ 7 vs. 8 [4 + 4 and 3 + 5] vs. 4 + 5 vs. primary 5) and PSA (≤ 20 vs. > 20 ng/ml). The Kaplan–Meier curves of the CRPC‐free rate stratified according to those categories are shown in Supplementary Figure S1 a‐c). Regarding the GS category, although we initially intended to compare four categories (GS ≤ 7 vs. 8 vs. 4 + 5 vs. primary 5), because univariate and multivariable Cox proportional hazards models revealed that the HR of the GS category of 8 for CRPC progression was similar to that of ≤ 7 (Supplementary Table S1), these two categories were integrated into one category (GS ≤ 8) and GS was regrouped into three categories: GS ≤ 8 vs. 4 + 5 vs. primary 5 (Supplementary Figure S1 d). Univariate and multivariable Cox regression analysis suggested that the risk of CRPC progression increased as the age at IMRT initiation decreased (Supplementary Table S1). Consequently, age was grouped into three categories (age ≤ 65 vs. > 65 and ≤ 75 vs. > 75), maintaining balance in the distribution among categories. The Kaplan–Meier curves of the CRPC‐free rate stratified according to these three age categories are shown in Supplementary Figure S1 e).

As the second process, discovery (n = 300) and validation (n = 300) cohorts were created using random assignment with the SURVEYSELECT procedure.

**FIGURE 1:**
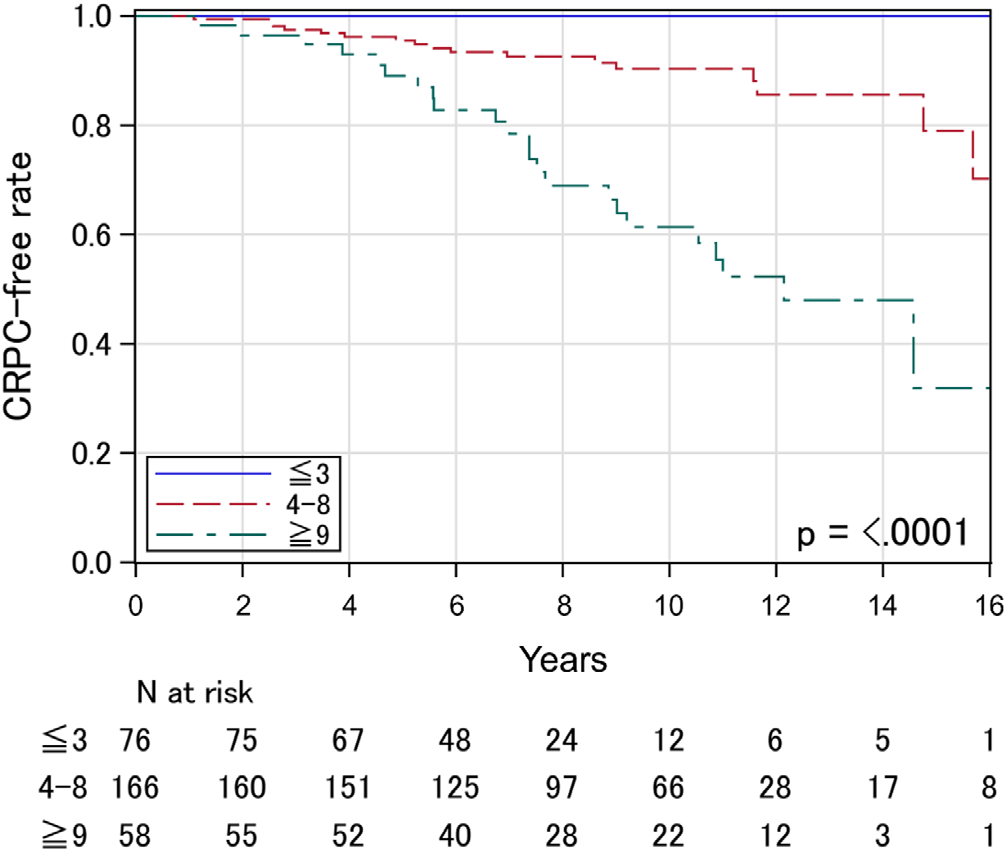
Castration‐resistant prostate cancer‐free rate stratified according to the novel risk model among patients in the discovery cohort.

As the third process, the cut‐off values of RS between the three risk categories of the current model (low‐, intermediate‐ and high‐risk) were determined. Table 2 shows the results of the multivariable Cox proportional hazards model with categorized covariables created in the first process among the discovery cohort: T‐stage (≤ T2c vs. T3a vs. ≥ T3b), GS (≤8 vs. 4 + 5 vs. primary 5), PSA (≤ 20 vs. > 20 ng/ml) and age (≤ 65 vs. > 65 and ≤ 75 vs. > 75 years old). In this process, we confirmed that the risk of CRPC progression decreased with age advanced. Each risk covariable was assigned a score based on the coefficient in the Cox proportional hazards model. Details of the assigned scores are also shown in Table 2. RS, the sum of the assigned score, ranged from 0 to 19. Then, based on the result of HR from univariate Cox proportional hazard models, low‐, intermediate‐ and high‐risk were defined as RS with 0–3, 4–8 and 9–19, respectively. Risk categories are summarized in Table 3. The Kaplan–Meier curves of these three risk categories displaying CRPC‐free rates showed wide separations in the discovery cohort (p < 0.0001) (Figure 1). Specifically, the 10‐year CRPC‐free rates were 100.0% (95% confidence interval [CI]: 100.0–100.0) in the low‐, 90.4% (95% CI: 83.8–94.4) in the intermediate‐ and 61.4% (95% CI: 45.5–73.9) in the high‐risk categories, respectively.

**TABLE 2:** Multivariable Cox proportional hazards model with categorized covariables for progression to CRPC in the discovery cohort, and details of the assignment scores.

	Coefficient (SE)	HR (95% CI)	*p* value	Assigned Score
Age (year‐old)				
≤ 65	0.79 (0.59)	2.21 (0.70–6.98)	0.18	3
> 65 and ≤ 75	0.46 (0.57)	1.58 (0.52–4.86)	0.42	2
> 75	0.00	1.00	ref	0
T stage				
≤ T2c	0.00	1.00	ref	0
T3a	0.77 (0.57)	2.15 (0.70–6.57)	0.18	3
≥ T3b	2.13 (0.57)	8.45 (2.77–25.79)	< 0.001	9
GS				
≤ 7, 4 + 4, and 3 + 5	0.00	1.00	ref	0
4 + 5	0.45 (0.44)	1.56 (0.66–3.69)	0.31	2
primary 5	1.27 (0.51)	3.55 (1.30–9.65)	0.01	5
PSA (ng/mL)				
≤ 20	0.00	1.00	ref	0
> 20	0.54 (0.38)	1.71 (0.82–3.58)	0.15	2

In the assigned score calculation, 1 score each was assigned to those risk categories as every 0.25 increase in the coefficient (rounding off to the nearest integral number). Abbreviations: SE, standard error; HR, hazard ratio; GS, Gleason score; PSA, prostate‐specific antigen.

**TABLE 3:** Summary of the current novel risk score model.

	Assigned Score	Risk Score (sum of assigned score)
Age (year‐old)		
≤ 65	3	
> 65 and ≤ 75	2	
> 75	0	
T stage		
≤ T2c	0	
T3a	3	
≥ T3b	9	
GS		
≤ 7, 4 + 4, and 3 + 5	0	
4 + 5	2	
primary 5	5	
PSA (ng/mL)		
≤ 20	0	
> 20	2	
Risk category		
Low‐risk		0–3
Intermediate‐risk		4–8
High‐risk		9–19

Abbreviations: GS, Gleason score; PSA, prostate‐specific antigen.

### 3.3 Validation of the Risk Score Model

**FIGURE 2:**
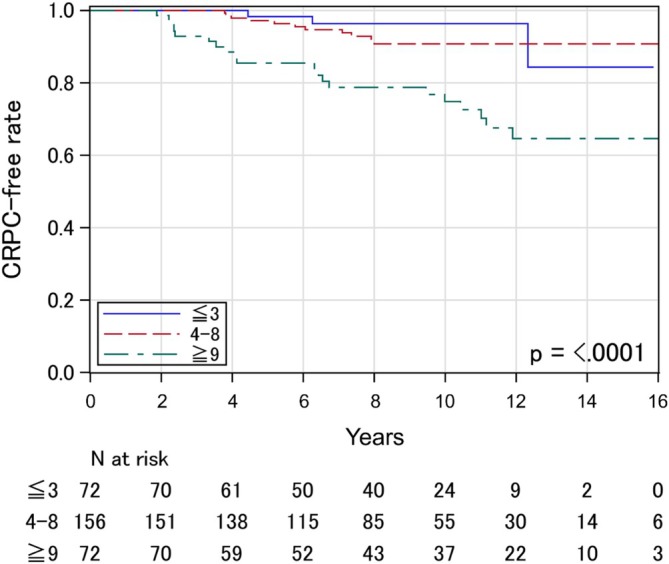
Castration‐resistant prostate cancer‐free rate stratified according to the novel risk model among patients in the validation cohort.

As the fourth process, this RS model was applied to the validation cohort (n = 300). Separation of the Kaplan–Meier curves of the CRPC‐free rate among these three risk categories was reproduced in the validation cohort (p < 0.0001) (Figure 2). Specifically, the 10‐year CRPC‐free rates were 96.4% (95% CI: 86.1–99.1) in the low‐risk, 90.7% (95% CI: 83.7–94.8) in the intermediate‐risk and 74.8% (95% CI: 62.0–83.8) in the high‐risk categories. Harrell's C‐index for this model was 0.692.

As an additional investigation, the current RS model was compared with NCCN risk classification using the validation cohort. Harrell's C‐index for NCCN risk classification was 0.617, being lower than that of the current RS model.

## 4 DISCUSSION

In this study, we developed a novel RS model to predict clinical outcomes following high‐dose IMRT for patients with high‐ and very high‐risk PCa. As components of our RS model, we included a novel risk factor, a younger age, as well as the three existing factors: clinical T‐stage, GS and PSA. In addition, we set CRPC progression as the endpoint, considered to be a promising surrogate for survival outcomes.^12^ Although a marked number of risk classifications for localized and locally advanced PCa have been proposed, most of those models use BF as an endpoint.^7^, ^8^, ^21^ Therefore, the accuracy of such previous models may be limited with regard to predicting survival outcomes. During the process of RS model development, we sub‐categorized NCCN high‐ and very high‐risk PCa into three risk classes (Tables 2 and 3). As a result of internal validation of this model, the reproducibility of the RS model was confirmed, and its Harrell's C‐index was 0.692, being higher than that of NCCN risk classifications (Harrell's C‐index = 0.617). To the best of our knowledge, this is the first study involving the development of an RS model to predict CRPC progression after definitive high‐dose IMRT for high‐ and very high‐risk PCa.

During the development of the current RS model, we determined the cut‐off values of each category (clinical T‐stage, GS and PSA) based on NCCN risk classifications. Regarding GS, as HR values were similar for GS sum ≤ 7 and GS sum 8 groups based on both univariate and multivariable analyses among all cases (n = 600), GS sum of ≤ 7 and GS sum of 8 were integrated into a single group. One possible explanation for this result is that we limited our study population only to patients with high‐ and very high‐risk PCa. Our results were in agreement with the observation in a retrospective analysis by Saad et al., in which GS was not significantly correlated with either BF‐free or distant metastasis‐free survival among 203 patients with high‐ and very high‐risk PCa who received definitive EBRT plus HT.^22^ Another explanation may be under‐estimation of GS due to sampling error via core needle biopsy. Concerning the age at the initiation of IMRT, as we confirmed a continuous decrease in the risk of CRPC progression with an increase of age at the initiation of IMRT in multivariable analysis including all cases (n = 600) (Supplementary Table S1), we divided the cohorts into three risk categories (age ≤ 65 vs. > 65 and ≤ 75 vs. > 75 years old).

In the current study, we included younger age as a risk factor in our RS model based on our previous observation, which revealed younger age (< 70 years old) as an independent predictive factor for both BF (HR: 1.69, 95% CI: 1.16–2.47, p = 0.006) and CRPC (HR: 2.58, 95% CI: 1.28–5.19, p = 0.008) in patients with non‐metastatic PCa treated with IMRT.^11^ Likewise, the nomogram predicting BF‐free survival following definitive EBRT by Gabriele et al. included younger age as a risk factor.^21^ This age dependency could be partly explained by the difference in time to testosterone recovery (TR). As time to TR has been reported to increase as age increases,^23^, ^24^ earlier TR in younger patients could contribute to a higher incidence of BF among such patients. However, regarding CRPC progression, which is the endpoint of our RS model, the difference in time to TR could not fully explain the reason for the higher incidence of CRPC progression among our younger patients, as CRPC progression occurs during HT. One possible explanation involves germline DNA‐repair gene mutations, such as BRCA2 gene mutation, which is detected more frequently in younger patients.^25^ However, given their relatively low prevalence, such mutations would be insufficient to solely explain the association between younger age and higher risk of CRPC progression. Another explanation would be that incomplete testosterone suppression during HT would lead to poorer clinical outcomes among younger patients. According to a study which investigated the association between an incomplete testosterone nadir and clinical outcomes among patients with localized PCa treated with definitive RT and HT, compared with patients with deeper nadir PSA (< 20 ng/dl), patients with shallow nadir PSA (20–49 ng/dl) showed significantly higher metastasis rates (subdistribution HR: 2.19, 95% CI: 1.21–3.94, p = 0.009) and a trend toward inferior prostate cancer‐specific mortality (subdistribution HR: 1.95, 95% CI: 0.90–4.22, p = 0.09).^26^ Incomplete testosterone suppression was reportedly frequently noted among younger patients (odds ratio: 0.95, 95% CI: 0.93–0.98, p = 0.001).^27^ Considering these findings together, although hypothetical, incomplete testosterone suppression in younger patients may lead to a higher incidence of CRPC progression. Thus, a treatment strategy to intensify HT, such as up‐front use of ARSIs, may be appropriate for younger patients. Further studies are needed to examine the association between younger age and higher risk of CRPC progression.

In the current study, patients categorized as low‐risk (RS of 0–3) achieved sufficient disease control with short‐term NA‐HT (median: 7.7 months) under the condition of the early initiation of salvage treatment after BF (trigger PSA: 4.0 ng/ml) (CRPC‐free rate: 96.4% at 10 years in the validation cohort). Although the addition of long‐term adjuvant A‐HT to definitive EBRT for patients with high‐ and very high‐risk PCa is recommended in several treatment guidelines,^2^, ^3^ the results suggest the possibility of shortening HT in this risk category under the condition of high‐dose EBRT and early initiation of salvage treatment after BF. For patients categorized as intermediate‐risk (RS of 4–8), there may remain room for improvement of tumour control, although CRPC‐free rates were considered favourable (CRPC‐free rate: 90.7% at 10 years). Therefore, the addition of long‐term A‐HT may be beneficial for this risk category, as recommended in the current treatment guidelines.^2^, ^3^ Conversely, tumour control of patients categorized as high‐risk (RS of 9–18) was markedly insufficient (CRPC‐free rate: 74.8% at 10 years). In addition, many of our patients categorized as high‐risk showed aggressive and rapid clinical courses. Specifically, 14.5% of those patients had progressed to CRPC within five years after IMRT despite the early initiation of salvage HT (Figure 2). Those results suggested the need for increased treatment intensity. Application of the up‐front use of ARSIs may be a promising strategy to improve the oncological outcomes of patients in our high‐risk category.^28^, ^29^

Our study had several limitations, including its retrospective nature of analysis. Firstly, our institutional HT method was unique. Although the addition of long‐term adjuvant A‐HT to definitive EBRT for patients with high‐ and very high‐risk PCa has been established as a standard in several treatment guidelines,^2^, ^3^ our patients solely received short‐term NA‐HT (median: 7.3 months), and salvage HT was added in the early phase after BF (trigger PSA: 4.0 ng/ml). Therefore, our results may not be directly applicable to patients with high‐ and very high‐risk PCa treated based on the current standard of care. However, this HT method was suggested to be a promising alternative method to the uniform administration of long‐term A‐HT. According to a Japanese phase III trial, which compared the effectiveness of two different HT methods in definitive EBRT for T3‐4N0M0 patients with PCa, non‐metastatic CRPC‐free survival rates did not differ significantly between a group receiving medium‐term HT (14 months) based on the policy of the early initiation of salvage HT (PSA > 5.0 ng/ml) (test arm) and a group receiving long‐term HT (5 years) (control arm) (HR: 1.13, 95% CI: 0.74–1.72, p = 0.56).^30^ Our HT method was similar to that of the test arm of this phase III study, although there were some differences in the radiation dose (78 vs. 72 Gy, respectively), period of A‐HT (0 vs. 6 months, respectively) and trigger PSA value for the initiation of salvage HT (4.0 vs. 5.0 ng/ml, respectively). Therefore, our clinical data may be used for the evaluation of CRPC progression outcomes, although we acknowledge that our data would not completely compensate the gap of the results between two HT methods. Secondly, we only performed internal validation; therefore, external validation is essential to support the reproducibility of our RS model. Thirdly, as our cohort included patients who were treated before the concept of CRPC became clearly established in 2008 by the Prostate Cancer Working Group 2.^31^ Therefore, the definition of CRPC in the current study was not exactly the same as that of the Prostate Cancer Working Group 3, which is currently used.^18^ Despite those limitations, our RS model was created using long follow‐up clinical data (median: 9.1 years). Furthermore, our novel RS model can be readily applied in daily clinical practice because it does not require additional examinations, such as molecular tests. Therefore, we consider that our study provides useful information for clinicians to individualize and optimize HT, including the application of the up‐front use of ARSIs. Given the marked heterogeneity of high‐ and very high‐risk PCa, the insights gained from our study are of particular importance.

In conclusion, we developed a novel RS model to predict CRPC progression among patients with high‐ and very high‐risk PCa treated with high‐dose IMRT. This RS model may provide useful information to tailor treatment intensity for this heterogeneous population. Further investigations including external validation of this model and establishment of an optimal HT strategy with the addition of high‐dose RT are warranted.

## AUTHOR CONTRIBUTIONS

Takashi Ogata performed the research; Takashi Ogata, Rihito Aizawa, Hiroyasu Abe, Satoshi Morita and Takashi Mizowaki designed the research study.

**Takashi Ogata:** conceptualization, data curation, formal analysis, investigation, methodology, writing—original draft. **Rihito Aizawa:** conceptualization, data curation, formal analysis, funding acquisition, investigation, methodology, project administration, supervision, writing—supervision of original draft, review and editing. **Hiroyasu Abe:** conceptualization, methodology, formal analysis, writing—review and editing. **Takayuki Goto:** investigation, writing—review and editing. **Kiyonao Nakamura:** iInvestigation, writing—review and editing. **Yuki Kita:** investigation, writing—review and editing. **Takayuki Sumiyoshi:** investigation, writing—eview and editing. **Kaoru Murakami:** investigation, writing—review and editing. **Kei Mizuno:** investigation, writing—review and editing. **Satoshi Morita:** conceptualization, methodology, formal analysis, writing—review and editing. **Takashi Kobayashi:** investigation, supervision, writing—review and editing. **Takashi Mizowaki:** conceptualization, funding acquisition, investigation, methodology, project administration, supervision, writing—review and editing.

## CONFLICT OF INTEREST STATEMENT

Takashi Mizowaki serves as a consultant to Novartis.

Takashi Mizowaki received a research grant from BrainLab AG, Hitachi High Tech and Varian Medical Systems.

Takashi Mizowaki received payment or honoraria for lectures, presentations, speakers bureaus, manuscript writing or educational events from Brainlab AG, Chugai Pharmaceutical, Eisai, Elekta, Eli Lilly, Ferring, Hitachi Ltd., Janssen, MC Medical, Nippon Kayaku, Novartis, Takeda and Varian Medical Systems.

Takashi Mizowaki has a leadership or fiduciary role in the Japanese Society for Radiation Oncology, Japan Society of Clinical Oncology, Japan Society of Urologic Oncology and Japan Radiological Society.

Takashi Kobayashi serves as a consultant to Astellas Pharma, AstraZeneca, Bayer, Chugai Pharmaceutical, Medicaroid, Merck & Co. and Janssen.

Takashi Kobayashi received a research grant from Astellas Pharma, Bayer, Chugai Pharmaceutical and Merck & Co.

Takashi Kobayashi received payment or honoraria for lectures, presentations, speakers bureaus, manuscript writing or educational events from Astellas Pharma, AstraZeneca, Bayer, Bristol Myers Squibb, Ono Pharmaceutical, Chugai Pharmaceutical, Janssen, Kissei Pharmaceutical, Merck & Co., Pfizer and Takeda.

Satoshi Morita received lecture fees from MSD.

## Supporting information


**Table S1.** Univariate and multivariable Cox proportional hazards model for progression to CRPC in all patients.

**Figure S1.** Castration‐resistant prostate cancer‐free rate in all patients stratified according to (a) T‐stage (≤ T2c vs. T3a vs. ≥ T3b), (b) Gleason score (≤ 7 vs. 8 [4 + 4 and 3 + 5] vs. 4 + 5 vs. primary 5), (c) PSA (≤ 20 vs. > 20 ng/ml), (d) Gleason score (≤ 8 vs. 4 + 5 vs. primary 5) and (e) age at IMRT initiation (age ≤ 65 vs. > 65 and ≤ 75 vs. > 75 year‐old).
