# Supplementary material for: Development of a novel risk model to predict CRPC progression following IMRT: Implications for tailoring treatment intensity
Source: BJUI Compass. 2025 Sep 7;6(9):e70074. doi: 10.1002/bco2.70074 (PMC12415265; doi:10.1002/bco2.70074)
Supplement: Supplementary file 1 — Table S1. Univariate and multivariable Cox proportional hazards model for progression to CRPC in all patients. [file BCO2-6-e70074-s002.docx]

Supplementary Table S1. Univariate and multivariable Cox proportional hazards model for progression to CRPC in all patients

|  | Univariate analysis | | |  | Multivariable analysis | | |
| --- | --- | --- | --- | --- | --- | --- | --- |
|  | Coefficient (SE) | HR (95% CI) | *p* value |  | Coefficient (SE) | HR (95% CI) | *p* value |
| Age (every five-year) | -0.25 (0.09) | 0.78 (0.66–0.92) | 0.004 |  | -0.23 (0.09) | 0.79 (0.67–0.94) | 0.008 |
| T stage |  |  |  |  |  |  |  |
| ≤ T2c | 0.00 | 1.00 | ref |  | 0.00 | 1.00 | ref |
| T3a | 0.80 (0.38) | 2.23 (1.06–4.69) | 0.04 |  | 0.80 (0.39) | 2.21 (1.04–4.72) | 0.04 |
| ≥ T3b | 1.96 (0.38) | 7.13 (3.41–14.90) | < 0.001 |  | 1.91 (0.39) | 6.76 (3.16–14.48) | < 0.001 |
| GS |  |  |  |  |  |  |  |
| ≤ 7 | 0.00 | 1.00 | ref |  | 0.00 | 1.00 | ref |
| 4+4 and 3+5 | -0.11 (0.30) | 0.89 (0.50–1.60) | 0.70 |  | 0.00 (0.30) | 1.00 (0.56–1.80) | 1.00 |
| 4+5 | 0.55 (0.31) | 1.73 (0.94–3.18) | 0.08 |  | 0.34 (0.32) | 1.41 (0.76–2.63) | 0.28 |
| primary 5 | 1.02 (0.38) | 2.79 (1.34–5.81) | 0.006 |  | 1.39 (0.38) | 4.03 (1.90–8.53) | < 0.001 |
| PSA (ng/mL) |  |  |  |  |  |  |  |
| ≤ 20 | 0.00 | 1.00 | ref |  | 0.00 | 1.00 | ref |
| > 20 | 0.74 (0.26) | 2.10 (1.27–3.46) | 0.004 |  | 0.49 (0.27) | 1.63 (0.97–2.74) | 0.07 |

Abbreviations: SE, standard error; HR, hazard ratio; GS, Gleason score; PSA, prostate-specific antigen.
