# Supplementary figures and images for: Development of a novel risk model to predict CRPC progression following IMRT: Implications for tailoring treatment intensity
Source: BJUI Compass. 2025 Sep 7;6(9):e70074. doi: 10.1002/bco2.70074 (PMC12415265; doi:10.1002/bco2.70074)

Figure S1 a.


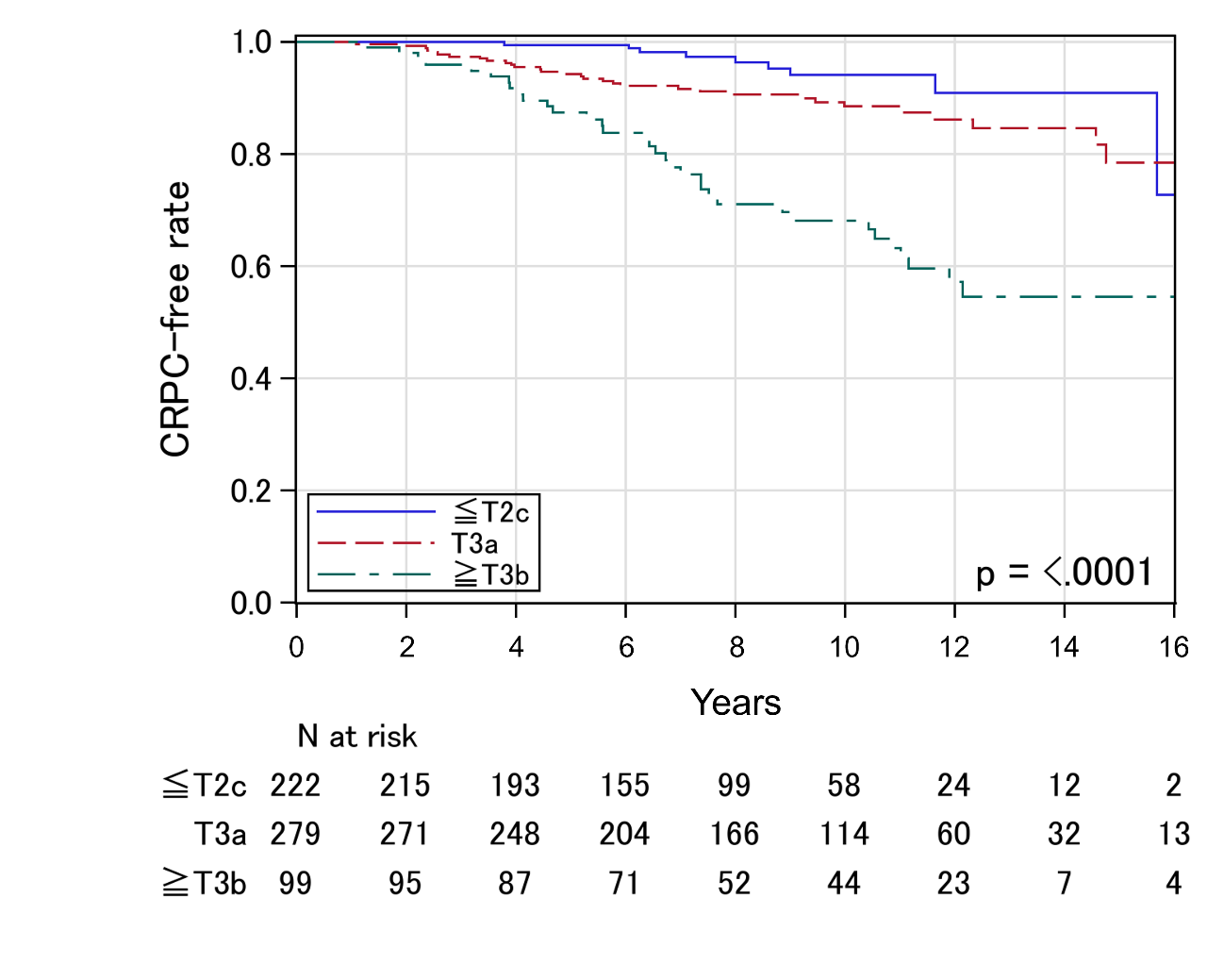


Figure S1 b.


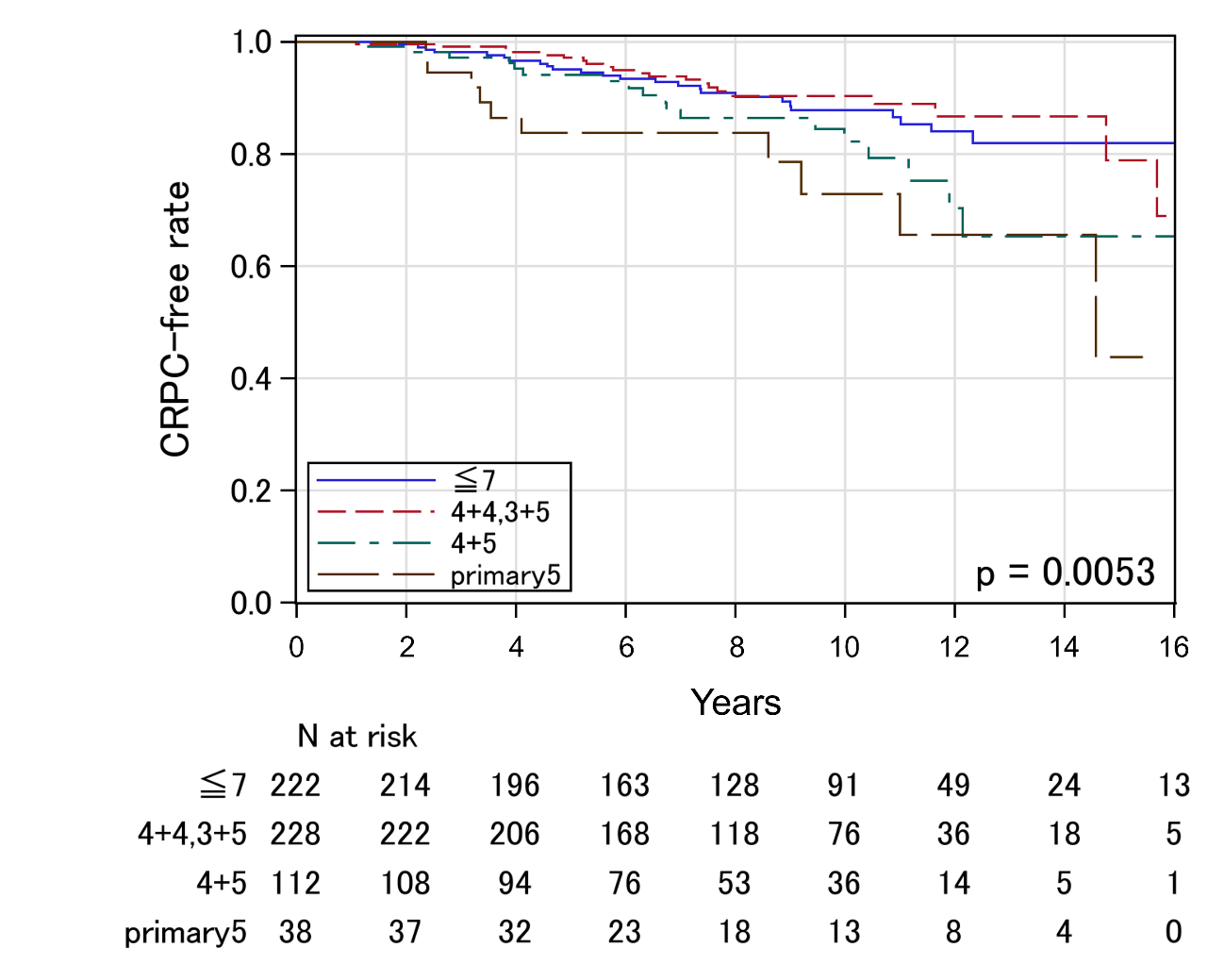


Figure S1 c.


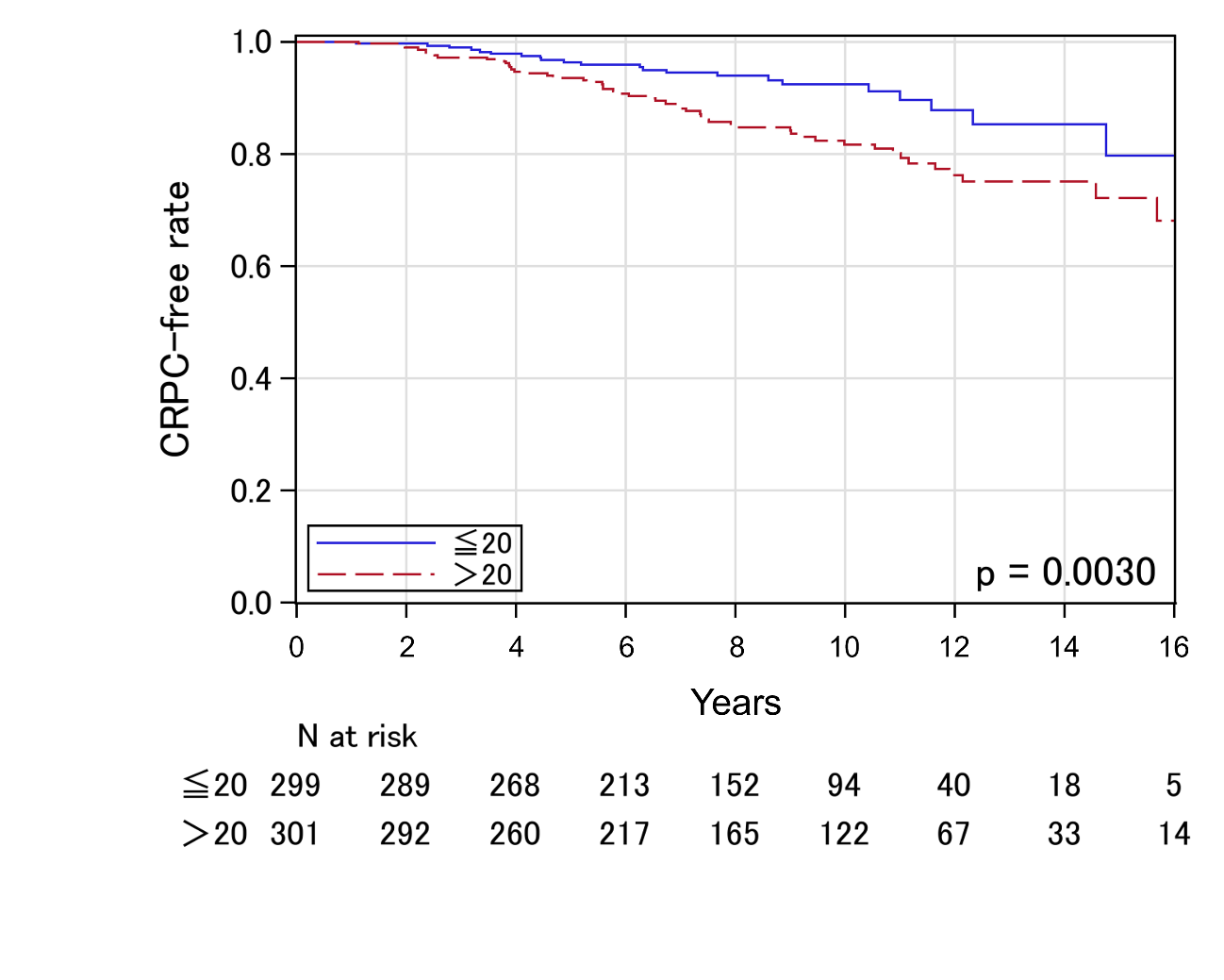


Figure S1 d.


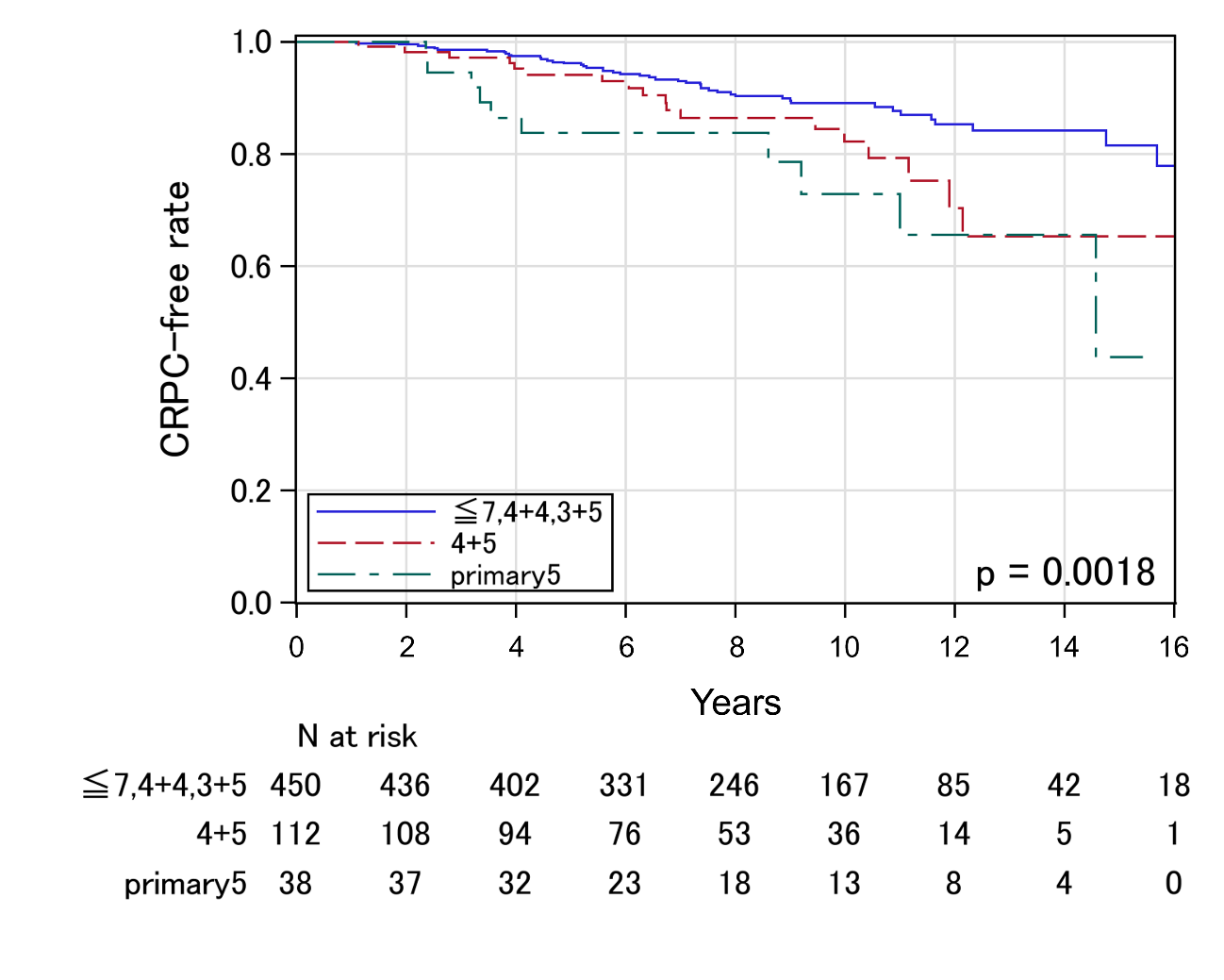


Figure S1 e.


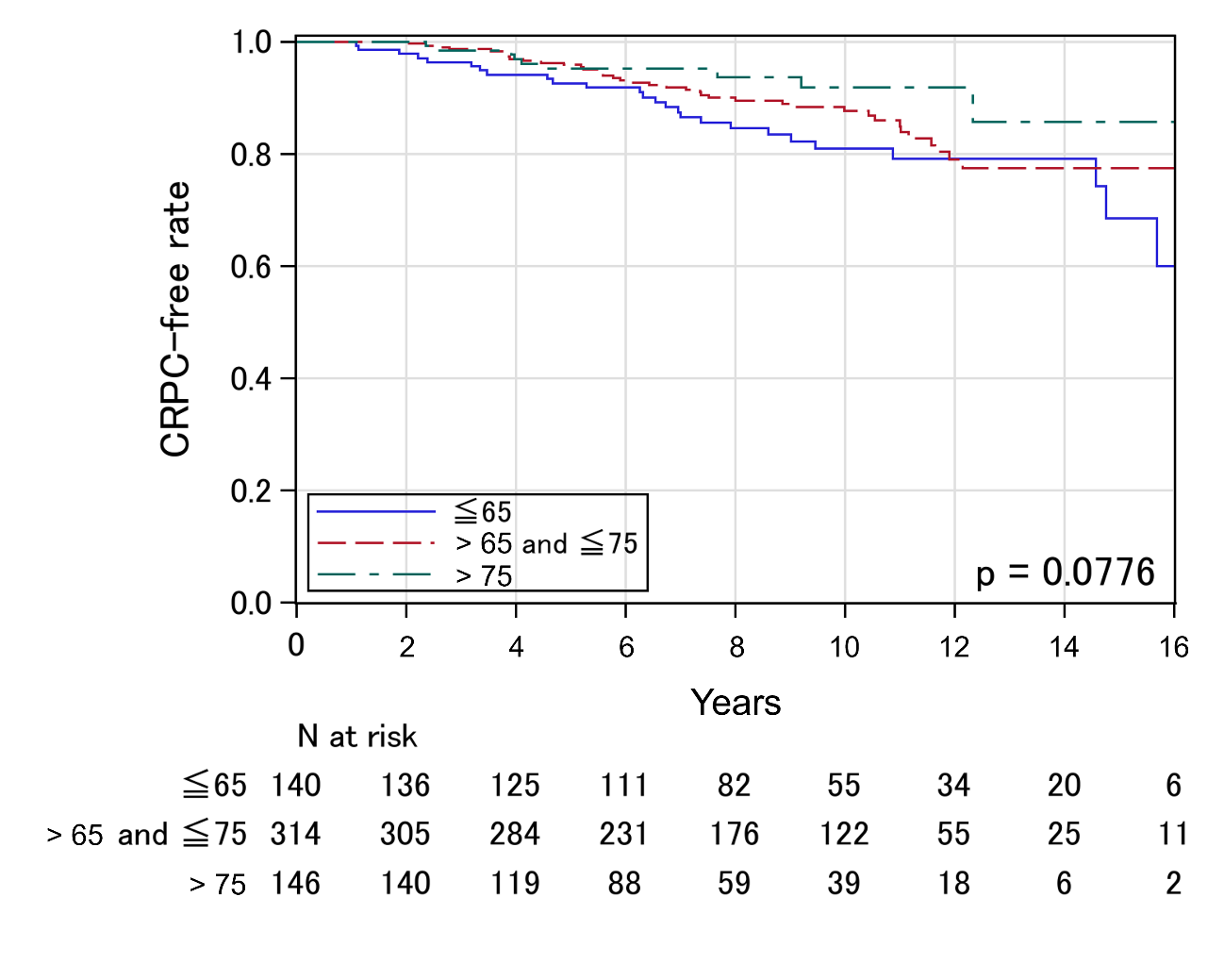

Supplement: Supplementary file 2 — Figure S1. Castration‐resistant prostate cancer‐free rate in all patients stratified according to (a) T‐stage (≤ T2c vs. T3a vs. ≥ T3b), (b) Gleason score (≤ 7 vs. 8 [4 + 4 and 3 + 5] vs. 4 + 5 vs. primary 5), (c) PSA (≤ 20 vs. > 20 ng/ml), (d) Gleason score (≤ 8 vs. 4 + 5 vs. primary 5) and (e) age at IMRT initiation (age ≤ 65 vs. > 65 and ≤ 75 vs. > 75 year‐old). [file BCO2-6-e70074-s001.docx]
